# Trends in racial/ethnic disparities in medical and oral health, access to care, and use of services in US children: has anything changed over the years?

**DOI:** 10.1186/1475-9276-12-10

**Published:** 2013-01-22

**Authors:** Glenn Flores, Hua Lin

**Affiliations:** 1 Division of General Pediatrics, Department of Pediatrics, UT Southwestern Medical Center, 5323 Harry Hines Blvd, Dallas, TX 5390-9063, USA; 2 Division of General Pediatrics, Children’s Medical Center, 1935 Medical District Dr, Dallas, TX, 75235, USA

**Keywords:** Disparities, Minorities, Children, Race, Ethnicity, Blacks, Hispanics, Asians/Pacific islanders, Native Americans, Multiracial

## Abstract

**Introduction:**

The 2010 Census revealed the population of Latino and Asian children grew by 5.5 million, while the population of white children declined by 4.3 million from 2000-2010, and minority children will outnumber white children by 2020. No prior analyses, however, have examined time trends in racial/ethnic disparities in children’s health and healthcare. The study objectives were to identify racial/ethnic disparities in medical and oral health, access to care, and use of services in US children, and determine whether these disparities have changed over time.

**Methods:**

The 2003 and 2007 National Surveys of Children’s Health were nationally representative telephone surveys of parents of 193,995 children 0-17 years old (N = 102,353 in 2003 and N = 91,642 in 2007). Thirty-four disparities indicators were examined for white, African-American, Latino, Asian/Pacific-Islander, American Indian/Alaskan Native, and multiracial children. Multivariable analyses were performed to adjust for nine relevant covariates, and Z-scores to examine time trends.

**Results:**

Eighteen disparities occurred in 2007 for ≥1 minority group. The number of indicators for which at least one racial/ethnic group experienced disparities did not significantly change between 2003-2007, nor did the total number of specific disparities (46 in 2007). The disparities for one subcategory (use of services), however, did decrease (by 82%). Although 15 disparities decreased over time, two worsened, and 10 new disparities arose.

**Conclusions:**

Minority children continue to experience multiple disparities in medical and oral health and healthcare. Most disparities persisted over time. Although disparities in use of services decreased, 10 new disparities arose in 2007. Study findings suggest that urgent policy solutions are needed to eliminate these disparities, including collecting racial/ethnic and language data on all patients, monitoring and publicly disclosing disparities data annually, providing health-insurance coverage and medical and dental homes for all children, making disparities part of the national healthcare quality discussion, ensuring all children receive needed pediatric specialty care, and more research and innovative solutions.

## Introduction

2010 Census data reveal that there not only has been a higher-than-anticipated growth in racial/ethnic minority populations of US children since 2000, but that it is highly likely that minority children will outnumber white children by the 2020 Census [1]. From 2000-2010, the population of white children in America declined by 4.3 million, whereas Latino and Asian/Pacific Islander (API) children increased by 5.5 million [1]. Indeed, all of the growth of the child population in America from 2000 to 2010 is attributable to population increases in children who are Latino, API, multiracial, or self-identified as “some other race” besides white [1].

Analyses of nationally representative data document that, compared with white children, racial/ethnic minority children in the US experience multiple disparities in medical and oral health, access to care, and use of services [2,3]. Nevertheless, very little is known about whether such disparities in medical and oral health and healthcare persist over time, and whether disparities can arise anew, worsen, improve, or be eliminated with time. A recent systematic review [4] of 56 years of the literature revealed that racial/ethnic disparities in children’s health and healthcare are extensive, pervasive, and occur across the spectrum of health and healthcare, but only four published studies were identified which examined disparities trends over times, and these analyses specifically only examined secular trends in overall [5] or congenital heart-defect [6] mortality rates, elevated blood-lead concentrations [7], or asthma prevalence, mortality, and hospitalizations [8].

The goals of this study, therefore, were to examine secular trends in racial/ethnic disparities in medical and oral health, access to care, and use of services in US children, and to identify which disparities arise anew, worsen, improve, or are eliminated over time.

## Methods

### Data sources

The 2003 National Survey of Children’s Health (NSCH) and 2007 NSCH are nationally representative surveys conducted by telephone in English and Spanish in 2003, and in English, Spanish, Mandarin, Cantonese, Vietnamese, and Korean in 2007 [9,10]. NSCH examines children’s physical, emotional, and behavioral health; family context; and the neighborhood environment. Data were collected using the State and Local Area Integrated Telephone Survey program. A random-digit-dialed sample of households with children <18 years old was selected from each of the 50 states and the District of Columbia. NSCH sampling was conducted to generate weighted estimates to represent the population of non-institutionalized children 0-17 years old nationally and in each state. Sampling weights also adjust for households with multiple telephone lines and non-response bias, including for unknown household status and eligibility, households with multiple children, and households without telephones.

A total of 102,353 interviews were completed for the 2003 NSCH, and 91,642 interviews for the 2007 NSCH. The weighted overall NSCH response rates were 55.3% in 2003 and 46.7% in 2007. API and American Indian/Alaska Native (AIAN) samples were not nationally representative in publicly available NSCH data, to protect confidentiality. Thus, to obtain nationally representative estimates for all indicators, non-public data were accessed and analyzed at the National Center for Health Statistics.

As this study consisted solely of analysis of secondary data, it was classified as exempt from Institutional Review Board review.

### Analyses

SAS 9.1© (Cary, NC) was used for all analyses. To examine secular trends in racial/ethnic disparities, analyses were performed comparing the 2007 and 2003 NSCH waves. The analytic framework consisted of identifying 1) disparities in the most recently available data (2007 NSCH), 2) the number of disparities for at least one group and overall in 2003 and 2007, and whether significant changes occurred; and 3) which disparities in 2007 were new, worsened, improved, or eliminated, compared with 2003. Consistent with prior NSCH disparities studies [2,3], analyses focused on comprehensive examination of three major disparities outcome domains: medical and oral health status, access to medical and dental care, and use of medical and dental services. Specific outcome measures included 22 for medical and oral health status (including developmental delays/problems and special healthcare-needs conditions), nine for access to medical and dental care, and three for use of medical and dental services. Bonferroni adjustments were not made, consistent with published guidelines [11] and prior NSCH disparities analyses [2,3], because a specific a priori hypothesis was tested for each dependent variable. Although one NSCH question addressed interpreter availability, only bivariate analyses could be performed for this outcome, due to limited sample sizes.

The main independent variable was children’s race/ ethnicity, classified based on parental response as white, African-American/black, Latino/Hispanic, API, AIAN, or multiracial (defined as any combination of two or more of the five aforementioned racial/ethnic groups). Additional demographic variables analyzed included the child’s age, gender, and insurance coverage, the number of children and adults in the household, the highest educational attainment in the household, household employment status, the primary language spoken at home, and combined annual family income. Consistent with prior analyses of disparities in children’s health and healthcare, these eight variables served as the covariates adjusted for in all multivariable analyses (except for insurance coverage when it was the dependent variable). The child’s insurance coverage was categorized as uninsured, or publicly or privately insured, based on coverage at the time of survey administration. Highest household educational attainment was dichotomized as high-school graduate or greater versus not a high-school graduate. Household employment was coded affirmatively if anyone in the household was employed for ≥50 weeks in the prior year. Combined annual family income was dichotomized as < vs. ≥100% of the federal poverty threshold (FPT) at the time of the survey.

SUDAAN^©^ was used to adjust for NSCH’s complex survey design. Pearson’s Chi-square test was used to examine associations between race/ethnicity and categorical variables, and the t-statistic for disparities in means of continuous variables. To account for the complex survey design, the statistic was turned into an F-statistic with non-integer degrees of freedom using a second order Rao and Scott correction. A two-tailed *P* < .05 was considered statistically significant.

To adjust for relevant covariates using a model maximizing fit, stepwise multivariable logistic regression was conducted, forcing the aforementioned eight covariates (the child’s age and insurance coverage, the number of children and adults in the household, the highest educational attainment in the household, household employment status, the primary language spoken at home, and combined annual family income), and using an initial alpha-to-enter of 0.15. For each indicator, adjusted odds ratios with 95% confidence intervals are reported for each racial/ethnic minority group, compared with white children. To examine disparity secular trends, z-scores and their associated *P* values were used to identify statistically significant changes in adjusted odds ratios between the 2003 and 2007 NSCH waves.

## Results

In reporting the results, the 2007 NSCH findings are presented by specific racial/ethnic groups, followed by secular trends between the 2003 and 2007 NSCH. Results of analyses of the 2003 NSCH are provided in Additional File 1: Appendices 1 and 2.

### Sociodemographic characteristics

#### Non-financial characteristics

Compared with white children, multiracial, API, Latino, and AIAN children were younger, and African-Americans slightly older (Table 1). Only 3% of white children live in households in which the highest educational attainment was not graduating high school, vs. over one-quarter of Latino children and over 10% of AIAN and African-American children. Latino, API, and AIAN children were significantly more likely than white children to reside in households in which English was not the primary language spoken at home. Compared with white households, AIAN, Latino, and African-American households are more likely to have >3 children, and all minority households are more likely to have >2 adults and adult unemployment.

**Table 1:** **Selected sociodemographic characteristics of US children 0**-**17 years old** (**N** = **90**,**117**) **by race**/**ethnicity** (**2007 NSCH**)

	**Mean or Proportion for**						
**Characteristic**	**Whites N** = **61**,**377**	**Latinos N** = **11**,**523**	**African**-**Americans N** = **8**,**873**	**Asians**/**PacificIsl anders N** = **2**,**771**	**American Indians**/ **Alaska Natives N** = **1**,**244**	**Multiracial Children N** = **4**,**329**	***P***
Mean age (yrs)	8.8	8.1	9.1	7.8	8.5	7.6	<.0001
Male gender (%)	51.8	51.0	50.1	46.1	53.3	49.6	.21
Highest educational attainment in household (%)							<.0001
Not a high-school graduate	3.3	26.7	10.4	3.6	11.3	4.8	
High-school graduate	19.2	31.3	32.1	12.4	34.4	21.8	
At least some college	77.5	42.0	57.6	84.0	53.4	73.4	
Primary language spoken at home not English (%)	1.0	52.1	1.9	40.9	3.8	1.3	<.0001
Number of children in household (%)							<.0001
1	23.6	16.4	27.4	28.6	28.0	27.2	
2	41.3	35.6	33.4	45.8	34.0	36.8	
3	25.6	33.4	25.4	18.3	22.9	25.3	
>3	9.5	14.6	13.7	7.3	15.0	10.8	
Number of adults in household (%)							<.0001
1	8.5	9.6	28.1	5.1	19.7	14.7	
2	75.4	61.4	49.8	68.1	56.9	65.9	
>2	16.1	29.0	22.1	26.8	23.4	19.4	
Adult in household employed ≥50 weeks in past year (%)	93.0	79.1	82.5	89.4	79.9	89.1	<.0001
Combined family income: % of federal poverty threshold (%)							<.0001
<100%	8.1	31.4	28.7	9.0	30.1	15.9	
100%-199%	15.7	26.1	25.6	13.1	22.9	22.7	
200% - 299%	18.7	12.9	14.5	10.9	14.0	15.6	
300% - 399%	15.3	7.2	8.9	13.4	10.8	11.9	
≥400%	35.4	11.8	13.5	41.4	15.3	27.7	
Unknown	6.9	10.7	.8	12.2	7.0	.1	

Data source: non-public data set of the 2007 National Survey of Childhood Health.

#### Household income

Only 8% of white children are poor, compared with almost one-third of Latino and AIAN households, over one-quarter of African-American households, and 16% of multiracial households (Table 1). Over one half of Latino, African-American, and AIAN children live in low-income households (<200% of the FPT), compared with less than ¼ of white children. In contrast, over 1/3 of white and more than 40% of API households have a combined annual family income ≥ 400% of FPT, vs. 12-15% of Latino, African-American, and AIAN households, and about ¼ of multiracial households.

### Medical and oral health

#### Overall health status, overweight, and special needs

In bivariate analyses, minority children were significantly more likely than white children to not have excellent/very good health status (Table 2), with Latinos, African-Americans, APIs, and AIANs having a substantially higher prevalence of not excellent/very good health than whites. Almost ¼ of African-American, Latino, and AIAN children are obese, compared with 13% for white children and 10% for API children, and about 40% of African-American, Latino, and AIAN children are overweight, vs. 27% of white and 21% of API children. AIAN children had the highest prevalence of all racial/ethnic groups of needing more medical care than others; needing/getting special therapy; emotional, developmental, or behavioral problems needing treatment or counseling; and learning disabilities. Both AIAN and African-American children also had the highest prevalence of having limited abilities.

**Table 2:** **Medical and oral health status of US children 0**-**17 years old** (**N** = **90**,**117**) **by race**/**ethnicity** (**2007 NSCH**)

	**Mean or Proportion for Each Racial**/**Ethnic Group**						
**Medical**/**Oral Health Status Measure**	**White N** = **61377**	**Latino N** = **11523**	**African**-**American N** = **8873**	**Asian**/**Pacific Islander N** = **2771**	**American Indians**/**Alaska Natives N** = **1244**	**Multiracial N** = **4329**	***P***
Health status^a^ (%)							<.0001
Excellent	68.8	46.5	53.1	59.3	58.7	65.6	
Very good	22.2	21.9	27.4	26.0	26.8	22.3	
Good	7.3	23.6	15.2	11.7	10.8	9.7	
Fair	1.4	7.2	3.9	2.6	3.0	2.1	
Poor	0.3	0.9	0.5	0.3	0.7	0.4	
BMI^b^ class (%)							<.0001
Underweight	5.1	4.6	4.7	12.3	3.7	3.9	
Normal	68.1	54.5	54.2	66.6	58.1	62.1	
Overweight^c^	14.0	17.5	17.3	11.3	15.2	19.8	
Obese^d^	12.9	23.4	23.8	9.9	23.0	14.3	
Needs more medical care than others (%)	12.5	10.9	12.8	6.0	16.5	13.4	<.0001
Has limited abilities (%)	5.4	5.8	9.9	4.4	10.1	7.2	<.0001
Needs/gets special therapy^e^ (%)	7.2	6.7	7.9	3.8	9.0	7.0	.0008
Emotional, developmental, or behavioral problems needing treatment or counseling (%)	7.1	6.2	8.7	2.5	12.1	8.2	<.0001
Learning disability (%)	10.1	11.4	12.4	3.1	14.6	10.6	<0001
Asthma (%)	12.4	12.4	19.6	12.1	16.2	18.0	<0001
Hearing/vision problems (%)	4.7	4.6	3.4	2.9	5.3	5.0	.03
ADHD (%)	9.5	5.1	9.6	2.0	11.7	12.0	<.0001
Depression/anxiety (%)	6.8	5.8	5.2	1.7	10.7	8.8	.0001
Behavior problems (%)	4.0	3.7	7.7	1.9	9.4	4.4	<.0001
Bone/joint/muscle problems (%)	3.3	3.1	2.7	2.5	4.1	3.0	.45
Diabetes (%)	0.	0.4	0.6	0.2	1.2	0.4	.03
Developmental delay (%)	4.9	4.5	5.5	2.0	5.4	6.2	.01
Respiratory allergies (%)	18.6	10.9	16.8	11.5	14.7	19.3	<0001
Digestive allergies (%)	4.9	3.6	5.8	5.0	6.0	6.2	.02
Skin allergies (%)	11.5	9.5	18.6	10.7	10.1	15.4	<0001
Headaches (%)	5.5	4.1	6.2	2.7	8.2	6.4	.0013
Speech problems (%)	6.3	5.8	6.8	2.9	6.5	6.1	.12
≥ 3 ear infections in last 12 mos (%)	6.4	7.0	5.1	2.9	6.8	7.1	.0001
Teeth condition^a^ (%)							<.0001
Excellent	54.3	31.2	37.9	49.8	42.6	54.9	
Very good	26.2	18.1	24.7	29.3	24.5	22.1	
Good	15.2	31.1	28.2	20.7	25.6	18.2	
Fair	3.6	16.4	7.2	8.2	5.2	4.2	
Poor	0.8	3.2	2.1	2.0	2.1	0.7	

Data source: non-public data set of 2007 National Survey of Childhood Health.

^a^By parental report.

^b^BMI = body mass index; ^c^BMI = 85-94% for age and gender; ^d^BMI ≥95% for age and gender.

^e^Includes physical, occupational, or speech therapy.

#### Specific health conditions

Disparities were noted in specific health conditions (Table 2). Asthma prevalence was highest among African-American, multiracial, and AIAN children, with about one in five African-American children afflicted with asthma. ADHD was highest among multiracial and AIAN children, at 12%, and particularly low among API children, at 2%, compared with 10% in white children. Depression/anxiety and diabetes were highest in AIANs and lowest in APIs. AIANs and African-Americans had a markedly higher prevalence of behavior problems than whites. The prevalence of developmental delay was highest for multiracial children and lowest for APIs. The prevalence of skin allergies was highest for African-American and multiracial children.

#### Teeth condition

Suboptimal (not excellent/very good) teeth condition was noted in over half of Latinos, more than 1/3 of African-Americans, and almost 1/3 of AIANs and APIs, compared with only 20% in whites (Table 2). For the remaining measures, there either were no statistically significant intergroup differences, or the differences were statistically significant but slight.

### Access to care and use of services

#### Insurance and PCPs

Only six percent of white children are uninsured, compared with 19% of Latino, 13% of AIAN, and nine percent of African-American children (Table 3). Latinos, AIANs, and African-Americans are more likely than whites to be sporadically insured. Only 5% of white children lack a personal doctor or nurse, compared with 18% of AIANs, 14% of Latinos, 11% of African-Americans, and 7% of both multiracial children and APIs. Only 12% of white parents reported that their child’s PCP never or only sometimes spends enough time with them; this proportion was significantly higher for all minority groups, with Latinos (38%), African-Americans (31%), AIANs (30%), and APIs (28%) having a particularly high prevalence.

**Table 3:** **Access to medical and dental care and use of medical and dental services for US children 0**-**17 years old** (**N** = **90**,**117**) **by race**/**ethnicity** (**2007 NSCH**)

	**Mean or Proportion for each Racial**/**Ethnic Group**						
**Access or Use**-**of**-**Service measure**	**White N=61,377**	**Latino N=11,523**	**African**-**American N=8,873**	**Asian**/**Pacific Islander N=2,771**	**American Indian**/**Alaska Native N=1,244**	**Multiracial N=4,329**	***P***
Insurance coverage at time of survey (%)							<.0001
None	6.1	18.9	8.8	5.0	13.0	6.4	
Public	17.6	44.4	50.8	17.2	45.6	33.2	
Private	75.8	35.9	38.9	76.0	39.7	60.0	
Insured, type unknown	0.5	0.8	1.5	1.8	1.7	0.5	
Sporadically insured^a^ in past year (%)	8.3	16.9	14.2	8.2	14.3	9.9	<.0001
Has personal doctor or nurse (%)	95.5	85.8	88.8	93.0	81.5	92.9	<.0001
PCP never/sometimes spends enough time with child (%)	11.9	37.8	30.5	27.6	29.6	15.0	<.0001
Interpreter needed to speak with PCP (%)	6.8	38.0	7.9	4.6	8.6	22.4	<.0001
Received all needed medical care (%)	96.9	95.7	95.2	98.1	94.4	95.1	<.0001
Received all needed dental care (%)	97.5	96.8	96.9	99.1	94.4	96.1	<.0001
Any problem getting specialty care (%)	18.6	30.9	33.6	26.3	39.8	26.9	<.0001
No physician visit in last 12 months (%)	11.5	14.1	8.5	11.9	11.9	10.6	<.0001
No routine preventive dental visit in last 12 months (%)	23.2	33.1	25.1	28.4	22.9	28.0	.0001
Received specialty care (%)	25.8	16.7	18.4	16.4	17.1	22.6	<.0001
Received mental healthcare in past 12 months (%)	9.1	6.1	7.9	2.7	12.2	10.1	.0001

Data source: non-public data set of the 2007 National Survey of Childhood Health.

^a^Defined as having but then losing health insurance at any time during the past 12 months.

#### Unmet needs and use of services

Only seven percent of white parents reported needing an interpreter to communicate with a PCP, compared with over 1/3 of Latino and almost ¼ of multiracial children (Table 3). AIANs, Latinos, African-Americans, and multiracial children were more likely than whites and APIs to have not received either all needed medical or dental care. Only 19% of whites experienced any problem obtaining specialty care, compared with 40% of AIANs, about one-third of African-Americans and Latinos, and over ¼ of APIs and multiracial children. Compared with other racial/ethnic groups, a higher proportion of Latinos and a lower proportion of African-Americans had no physician visit in the past year. About 1/3 of Latinos and over ¼ of APIs and multiracial children had no routine preventive dental visit in the past year, compared with ¼ or less for other racial/ethnic groups of children. Compared with white children, minority children were significantly less likely to receive specialty care. Lower proportions of APIs, Latinos, and African-Americans had received mental healthcare than AIANs, multiracial children, and whites.

### Multivariable analyses

#### Medical and oral health status

After multivariable adjustment for eight covariates, multiple racial/ethnic disparities persisted in medical and oral health status (Table 4). Compared with whites, Latinos, African-Americans, and multiracial children were significantly more likely to be in suboptimal (not excellent/ very good) health and to be overweight/obese. African-Americans were more likely than whites to have limited abilities and digestive allergies. Multiracial children, African-Americans, and Latinos were more likely than whites to have asthma. Multiracial children were more likely than whites to have ADHD and depression/anxiety. Compared with whites, AIANs and African-Americans had higher odds of behavior problems, and African-Americans and multiracial children had higher odds of skin allergies. All minority groups except for multiracial children were more likely than whites to have teeth in suboptimal condition. Of note, there were several medical and oral health indicators for which minority children had reduced odds compared with white children, including 12 for APIs, six for African-Americans, and one for Latinos.

**Table 4:** **Multivariable analyses**^**a**^**of racial**/**ethnic disparities in medical and oral health status among US children 0**-**17 years old** (**N** = **90**,**117**), **2007 NSCH**

	**Odds ratio** (**95**% **confidence interval**) **vs**. **white children**				
**Measure**	**Latino**	**African**-**American**	**Asian**/**Pacific Islander**	**American Indian**/**Alaska Native**	**Multiracial**
Health not excellent/very good	1.88 (1.56, 2.27)	1.78 (1.55, 2.04) Z = 2.01 (*P* = .02)	1.19 (0.84, 1.68)	1.15 (0.86, 1.54)	1.33 (1.05, 1.68)
Overweight or obese	1.60 (1.27, 2.02)	1.65 (1.43, 1.91)	0.64 (0.42, 0.99)	1.39 (1.00, 1.95)^c^	1.35 (1.06, 1.71)
Needs more medical care than others	0.89 (0.74, 1.06)	0.81 (0.69, 0.94)	0.51 (0.37, 0.71)	1.15 (0.83, 1.59)	1.04 (0.85, 1.28)
Has limited abilities	0.99 (0.75, 1.3)	1.36 (1.14, 1.62)	1.0 (0.64, 1.54)	1.4 (0.99, 1.98)	1.26 (0.96, 1.66)
Needs/gets special therapy^b^	0.94 (0.74, 1.21)	0.94 (0.78, 1.13)	0.59 (0.39, 0.9)	1.06 (0.73, 1.54)	0.89 (0.7, 1.15)
Emotional, developmental, or behavioral problems needing treatment or counseling	0.83 (0.66, 1.04) Z = -1.90 (*P* = .03)	0.84 (0.69, 1.01)	0.4 (0.27, 0.6)	1.35 (0.93, 1.97) Z = 1.69 (*P* = .045)	1.16 (0.92, 1.46)
Learning disability	1.12 (0.88, 1.43)	0.95 (0.79, 1.13)	0.34 (0.24, 0.5)	1.23 (0.84, 1.79)	1.05 (0.82, 1.36)
Asthmatic	1.28 (1.05, 1.56)	1.48 (1.29, 1.69)	1.32 (0.93, 1.88)	1.25 (0.93, 1.66)	1.59 (1.28, 1.97)
Hearing/vision problem	1.27 (0.9, 1.8)	0.60 (0.47, 0.75)	0.85 (0.47, 1.52)	0.96 (0.59, 1.57) Z = -2.29 (*P* = .01)	1.07 (0.71, 1.6)
ADHD	0.76 (0.57, 1.01)	0.76 (0.64, 0.91)	0.31 (0.21, 0.45)	1.06 (0.73, 1.54)	1.38 (1.06, 1.8) Z = 1.64 (*P* = .0499)
Depression/anxiety	0.91 (0.69, 1.21)	0.5 (0.39, 0.64)	0.31 (0.21, 0.48)	1.27 (0.8, 2.02)	1.38 (1.06, 1.81)
Behavior problems	0.95 (0.67, 1.34)	1.31 (1.06, 1.62)	0.31 (0.19, 0.53)	1.78 (1.07, 2.96) Z = 2.34 (*P* = .01)	1.06 (0.78, 1.45)
Bone/joint/muscle problems	1.03 (0.7, 1.52)	0.72 (0.54, 0.95)	0.87 (0.44, 1.72) Z = 2.63 (*P* = .004)	1.17 (0.65, 2.1)	0.98 (0.7, 1.36)
Diabetes	0.48 (0.24, 0.93)	0.99 (0.52, 1.88) Z = 1.66 (*P* = .048)	0.23 (0.05, 1.11)	0.29 (0.08, 1.07)	0.72 (0.37, 1.43)
Developmental delay	1.18 (0.85, 1.62) Z = 1.95 (*P* = .03)	0.88 (0.7, 1.1) Z = 1.90 (*P* = .03)	0.51 (0.34, 0.77)	0.88 (0.55, 1.39)	1.22 (0.86, 1.72)
Digestive allergies	1.09 (0.77, 1.53)	1.23 (1.01, 1.49)	1.18 (0.8, 1.73)	1.29 (0.77, 2.17)	1.21 (0.81, 1.82)
Skin allergies	1.16 (0.95, 1.4)	1.85 (1.63, 2.11)	1.1 (0.84, 1.45)	0.91 (0.64, 1.31) Z = -2.01 (*P* = .02)	1.35 (1.11, 1.64)
Speech problem	0.92 (0.7, 1.21)	0.91 (0.75, 1.11) Z = -2.91 (*P* = .002)	0.48 (0.27, 0.86)	0.88 (0.56, 1.38)	0.87 (0.67, 1.14)
≥ 3 ear infections in last 12 mos	0.93 (0.74, 1.16)	0.67 (0.55, 0.82)	0.42 (0.28, 0.62)	0.87 (0.6, 1.27)	0.91 (0.67, 1.22)
Teeth condition not excellent/very good	1.97 (1.68, 2.31) Z = 1.92 (*P* = .03)	1.87 (1.67, 2.08)	1.48 (1.15, 1.91)	1.45 (1.12, 1.88)	1.19 (0.96, 1.48)

Data source: Non-public data set of 2007 National Survey of Childhood Health.

^a^Adjusted for primary language spoken at home, child’s age and insurance coverage, caregiver’s educational attainment and employment status, number of children in the household, number of adults in the household, and poverty level. Z-scores and *P* values provided for ORs and 95% CIs which significantly differed for same indicator in multivariable analyses of 2003 NSCH (see Additional file 1: Appendices 1 and 2 for 2003 NSCH data).

^b^Includes physical, occupational, or speech therapy.

^c^Not statistically significant, but trending towards significance, with *P* = .0528.

#### Access to care and use of services

Multiple disparities also persisted in access to care and use of health services (Table 5). Compared with white children, AIANs, Latinos, and African-Americans were significantly more likely to be both uninsured and sporadically insured. AIANs had about four times the odds, and African-Americans, Latinos, and multiracial children about double the odds of whites of having no personal doctor or nurse. Parents in all minority groups were significantly more likely than whites to report the PCP never/sometimes spends enough time with the child. AIANs and multiracial children were twice as likely as whites to have not received all needed dental care. All minority groups except APIs were significantly more likely than whites to have had a problem obtaining specialty care. APIs, AIANs, and African-Americans were more likely to have received no specialty care in the past year. APIs had triple the odds and African-Americans double the odds of whites of receiving no mental healthcare in the past year. In contrast, there were only three indicators for which minority children had reduced odds compared with white children, including uninsurance and unmet dental needs for APIs, and physician visits in the past year for African-Americans.

**Table 5:** **Multivariable analyses**^**a**^**of racial**/**ethnic disparities in access to medical and dental care and use of services among US children 0**-**17 years old** (**N** = **90**,**117**), **2007 NSCH**

	**Odds ratio** (**95**% **confidence interval**) **vs**. **white children**				
**Measure**	**Latino**	**African**-**American**	**Asian**/**Pacific Islander**	**American Indian**/**Alaska Native**	**Multiracial**
No health insurance^b^	1.55 (1.24, 1.95)	1.33 (1.07, 1.65)	0.47 (0.33, 0.67)	1.97 (1.40, 2.79)	1.08 (0.82, 1.41)
Sporadically insured in past year^b^	1.72 (1.4, 2.11) Z = 1.79 (*P* = .04)	1.55 (1.31, 1.83)	0.96 (0.71, 1.3)	1.51 (1.07, 2.13)	1.11 (0.82, 1.51)
No personal doctor or nurse	1.71 (1.33, 2.21)	2.07 (1.73, 2.48)	1.42 (0.91, 2.21)	3.52 (1.91, 6.5)	1.55 (1.19, 2.01)
PCP never/sometimes spends enough time with child	2.2 (1.85, 2.61)	2.53 (2.25, 2.85)	2.46 (1.85, 3.26)	2.3 (1.75, 3.03)	1.28 (1.02, 1.62)
Did not receive all needed medical care	1.39 (0.95, 2.02)	1.23 (0.95, 1.6)	0.69 (0.45, 1.06)	1.43 (0.89, 2.3) Z = -2.4 (*P* = .01)	1.4 (0.82, 2.4) Z = -2.34 (*P* = .01)
Did not receive all needed dental care	1.15 (0.74, 1.8)	0.9 (0.64, 1.26) Z = -4.34 (*P* < .01)	0.42 (0.25, 0.72) Z = -2.45 (*P* = .01)	1.69 (1.05, 2.72)	1.5 (1.04, 2.18)
Any problem getting specialty care	1.43 (1.07, 1.92)	1.83 (1.45, 2.32) Z = 3.43 (*P* < .01)	1.48 (1.00, 2.19)^c^	2.34 (1.44, 3.79)	1.53 (1.01, 2.32)
No physician visit in last 12 months	1.01 (0.81, 1.25)	0.61 (0.51, 0.72) Z = -8.2 (*P* < .01)	1.28 (0.88, 1.85) Z = -2.53 (*P* = .01)	0.89 (0.61, 1.3) Z = -2.77 (*P* = .003)	1.0 (0.75, 1.34)
No routine preventive dental visit in last 12 months	1.05 (0.89, 1.25) Z = -1.76 (*P* = .04)	1.04 (0.9, 1.2) Z = -5.7 (*P* < .01)	1.08 (0.86, 1.35) sZ = -1.66 (*P* = .048)	0.7 (0.49, 1.0) Z = -3.5 (*P* < .01)	0.96 (0.77, 1.19) Z = -2.07 (*P* = .02)
Received no specialty care in last 12 months	1.14 (0.96, 1.35)	1.36 (1.20, 1.54)	1.59 (1.21, 2.07)	1.41 (1.04, 1.92)	1.12 (0.92, 1.36) Z = 1.93 (*P* = .03)
Received no mental healthcare in past 12 months	1.27 (1.00, 1.62)^d^	1.62 (1.33, 1.97)	2.83 (1.95, 4.1)	0.90 (0.59, 1.38)	0.9 (0.72, 1.14)

Data source: non-public data set of 2007 National Survey of Childhood Health.

^a^Except as noted below, adjusted for primary language spoken at home, child’s age and health insurance coverage, caregiver’s educational attainment and employment status, number of children in the household, number of adults in the household, and poverty level. NS = not statistically significant. Z-scores and *P* values provided for ORs and 95% CIs which significantly differed for same indicator in multivariable analyses of 2003 NSCH (see Additional file 1: Appendices 1 and 2 for 2003 NSCH data).

^b^Adjusted for primary language spoken at home, child’s age, caregiver’s educational attainment and employment status, number of children in the household, number of adults in the household, and poverty level.

^c^Not statistically significant, but trending towards significance, with *P* = .0504.

^d^Not statistically significant, but trending towards significance, with *P* = .0503.

### Secular trends in disparities between 2003 and 2007

The number of indicators for which at least one racial/ethnic group experienced disparities did not significantly change between 2003-2007, either overall, or in any of the three subcategories, at 22 vs. 18, respectively (Table 6). The total number of specific disparities in 2007 was 46, which was not a significant change from 2003, when there were 63 (*P* = .06); the only subcategory with a significant reduction (82%; *P* < .01) was use of services (Table 6).

**Table 6:** **Trends between the 2003 and 2007 NSCH in US children**’**s racial**/**ethnic disparities in medical and oral health**, **access to care**, **and use of services**

**Disparities category**	**Total number of NSCH indicators**^**a**^	**Disparities for at least one racial**/**ethnic minority group**		**Total number of disparities**^**b**^	
		**2003**	**2007**	**2003**	**2007**
Medical and oral health	22	10	10	26	20
Access to medical and dental care	9	9	7	26	24
Use of medical and dental services	3	3	1	11	2^c^
**TOTAL**	**34**	**22**	**18**	**63**	**46**

^a^Includes only those indicators available in both the 2003 and the 2007 NSCH.

^b^For each NSCH indicator, disparities were possible for up to all five racial/ethnic minority groups.

^c^*P* < .01 for comparison between 2003 and 2007.

Ten new disparities arose in 2007 which did not exist in 2003, including five for multiracial children (overweight, ADHD, depression/anxiety, PCP never/sometimes spends enough time with you, and problems getting specialty care), three for African-Americans (digestive allergies, uninsurance, and problems getting specialty care), and two for AIANs (teeth condition and behavior problems) (Table 7). Three disparities worsened between 2003 and 2007: teeth condition and sporadic insurance for Latinos, and no specialty care in the past year for AIANs. Two specific disparities improved over time (i.e., their odds ratios significantly decreased): health status for Latinos and not receiving all needed dental care for multiracial children. There were 26 disparities which were eliminated over time, including nine for AIANs and eight for multiracial children; almost 40% of these eliminated disparities (N = 10) were use-of-services indicators.

**Table 7:** **Specific racial**/**ethnic disparities in the medical and oral health and healthcare of US children which were new**, **worsened**, **improved**, **or eliminated between 2003 to 2007**

**Racial**/**ethnic minority group**	**New disparity in 2007 which did not exist in 2003**	**Disparity significantly worsened between 2003**-**2007**	**Disparity significantly improved between 2003**-**2007**	**Disparity eliminated between 2003**-**2007**
Latino	―	· Teeth condition	· Health status	· No specialty care in past year
		· Sporadic insurance		· No physician visit in past year
				· No routine dental visit in past year
African-American	· Digestive allergies	―	―	· Speech problem
	· Uninsured			· No physician visit in past year
	· Problems getting specialty care			· No routine preventive dental visit in past year
				· Did not receive all needed dental care in past year
Asian/Pacific Islander	―	―	―	· Problems getting specialty care
				· No physician visit in past year
American Indian/Alaska Native	· Teeth condition	· No specialty care in past year	―	· Overweight
	· Behavior problems			· Has limited abilities
				· Needs/gets special therapy
				· Asthma
				· Hearing/vision problems
				· Skin allergies
				· No physician visit in past year
				· No routine dental visit in past year
				· Did not receive all needed medical care
Multiracial	· Overweight	―	· Did not receive all needed dental care	· Teeth condition
	· ADHD			· Has limited abilities
	· Depression/anxiety			· Digestive allergies
	· PCP never/sometimes spends enough time with you			· Uninsured
				· Sporadic insurance
	· Problems getting specialty care			· No physician visit in past year
				· No routine dental visit in past year
				· Did not receive all needed medical care
**TOTAL**	**10**	**3**	**2**	**26**

## Discussion

### Disparities in 2007 NSCH

After adjustment for relevant covariates, multiple racial/ethnic disparities were documented in oral and medical health and healthcare. Despite increases in the numbers of pediatricians in the US, advances in medical care, the introduction of new antibiotics and other medications, and enhancements in the screening and detection of pediatric diseases during the past decade, the study findings revealed that Latino, African-American, and multiracial children are significantly more likely than white children to have suboptimal health, to be overweight, and to be afflicted with asthma. Elimination of such disparities may require targeted, tailored interventions which are community-based, leverage community health workers or parent mentors, and emphasize education and social support. For example, for minority children with asthma and their families, parent mentors (trained minority parents who already have children with asthma) have been shown to reduce wheezing, asthma exacerbations, ED visits, and missed parental work days, while improving parental self-efficacy, all at a reasonable cost, and with net cost savings [12].

#### Dental health

All racial/ethnic minority groups of children, except for multiracial children, are significantly more likely than white children to have teeth in suboptimal condition. The reasons for these disparities are unclear, and merit further study. For AIAN children, suboptimal oral health may be a result of their higher odds of unmet dental needs, but other minority groups did not differ from whites in unmet dental needs. No minority group differed from whites in the adjusted odds of no routine preventive dental visit in the past year, but this indicator only applies to children who have ever made a dental visit, so oral health disparities might relate to a greater risk of having never made a dental visit or later initiation of the first dental visit. Disparities in satisfaction with and the quality of dental care for minority children have been reported [13], and may also play a role in oral health disparities.

#### Insurance coverage

Latinos, AIANs, and African-Americans are significantly more likely than whites to be uninsured and sporadically insured, and these disparities persisted between 2003-2007, except that lack of health insurance arose as a new disparity in 2007 for African-American children. Latinos and African-Americans continue to be disproportionately represented among uninsured American children, with the two groups accounting for 40% of US children, but 57% of uninsured US children [14]. Not enough is known about why AIAN children continue to be at high risk for being uninsured and sporadically insured. Evidence from a randomized, controlled trial indicates that community health workers can eliminate racial/ethnic disparities in uninsurance [15], suggesting that great use of community health workers may be needed to achieve equity in health insurance coverage.

#### Especially marked disparities

Noteworthy disparities were identified in lacking a personal doctor or nurse (PDN), insufficient time with the child’s healthcare provider, and problems getting specialty care. All minority groups except for APIs have approximately double to triple the odds of having no PDN. Having a PDN―and the associated continuity of care― is a crucial component of the medical home, so many health and healthcare disparities identified in this study could relate to lack of a PDN and medical home. All minority groups had greater odds than whites of the PCP never/sometimes spending enough time with the child, which has the potential to result in poorer communication, lower patient/parent satisfaction, and possibly lower quality of care. Recently, it has been pointed out that racial/ethnic disparities in health and healthcare can be viewed as deficits in quality of care [16,17], and a comprehensive strategy using a quality-improvement framework and tailored interventions has been shown to reduce disparities [18]. All minority groups, except for APIs, have significantly higher odds than whites of experiencing problems getting specialty care. This is especially concerning, because inadequate access to pediatric specialists can result in poorer quality of care and adverse outcomes. For example, among minority children with asthma, only 17% were being seen by a pediatric asthma or pulmonary subspecialist, subspecialty care was associated with five times the odds of having an asthma care plan, and having an asthma care plan was associated with a significantly lower likelihood of asthma exacerbations in the past year [19].

#### Reverse differences

For 18 health and three use-of-services indicators, minority children had significantly lower odds than white children. The reasons behind these “reverse differences” are not entirely clear. It is possible that certain reverse differences—such as reduced odds of ADHD and of depression/anxiety among API and African-American children—may reflect underdiagnosis of these conditions due to the higher odds of the PCP never/sometimes spending enough time with the child and reduced access to specialty care and mental healthcare. For example, one study revealed that African-American children are less likely than white children to receive ADHD evaluation, diagnoses, and treatment [20]. In contrast, it is possible that other such reverse differences—such as reduced odds of overweight/obesity in APIs—may accurately reflect racial/ethnic variations in the prevalence of certain conditions. One systematic review, for example, concluded that Asian-Americans of all ages have a lower prevalence of obesity than all other racial/ethnic groups [21]. Reverse differences in use of services, such as lower odds vs. whites of having no physician visit in the past year for African-Americans or of not receiving all dental care for APIs, might potentially reflect better access to certain categories of healthcare for these racial/ethnic minority groups. Additional research clearly is needed on the reasons for such reverse differences and potential lessons that might be learned on how to enhance the health and healthcare of all children.

#### Disparities for specific racial/ethnic groups

Many substantial and noteworthy disparities varied by racial/ethnic group (Tables 4, 5, 7), and awareness of these particular disparities, almost all of which persisted over time, would seem to be crucial in their prevention and monitoring, as well as the development and implementation of interventions to reduce or eliminate these disparities. Latino children have especially high odds of suboptimal health status and teeth condition, overweight, lack of health insurance, and sporadic health insurance. African-American children have particularly high odds of overweight, limited abilities, asthma, behavior problems, skin allergies, having no personal doctor or nurse, the PCP never/sometimes spending enough time with the child, and problems getting specialty care. API children have especially high odds of the PCP never spending enough time with the child, receiving no specialty care, and receiving no mental healthcare. AIAN children have particularly high odds of behavior problems, no health insurance, no personal doctor or nurse, unmet dental needs, and problems getting specialty care. Multiracial children have especially high odds of asthma, ADHD, depression/anxiety, and unmet dental needs.

### Secular trends in disparities

#### Disparities reductions and eliminations

Between 2003 and 2007, some noteworthy progress was made in reducing and eliminating certain racial/ethnic disparities in children’s health and healthcare. A significant reduction occurred over time in the total number of disparities in medical and dental services, which dropped from 11 to two. Two specific disparities improved over time. In addition, 26 specific disparities were eliminated over time. Of note, there was elimination of disparities which existed in 2003 for all minority groups in having no physician visit in the past year and for four of five minority groups in having no routine preventive dental visit in the past year. This is the first study, to our knowledge, to report such positive secular trends in children’s disparities. The reasons for these trends are unclear, and warrant further study, with particular attention to such possibilities as greater parental awareness of the importance of routine medical and dental care, heightened attention to these disparities among health systems and clinicians, or changing school regulations for annual physical exams and vaccinations.

#### Persistent, worse, and new disparities

In contrast, no significant change over time was seen in the number of indicators for which at least one minority group experienced disparities, either overall, or in any of the three subcategories. Furthermore, neither the total number of specific disparities, nor the numbers of specific disparities in the medical/oral health and access to medical and dental care categories changed significantly over time. In addition, three disparities worsened and 10 new disparities arose over time. These findings indicate that much work remains to be done to eliminate racial/ethnic disparities and achieve equity in children’s medical and oral health and healthcare.

#### Importance of secular-trend analyses

The study findings underscore the importance of examining secular trends in disparities. For example, multiracial children experienced five new disparities over time during the study interval, while at the same time eight of their disparities were eliminated. These results suggest that the addition and elimination of disparities may be a continuously evolving process in a constant state of flux for certain groups, perhaps reflecting and acting in concert with changes in social determinants of medical and oral health and healthcare (such as poverty and educational opportunity), as well as changes in the healthcare system. For example, given that racial/ethnic minority children are disproportionately represented among the uninsured, changes over time in federal and regional poverty, temporary caps or freezes in state Medicaid and CHIP, out-of-pocket expenses, and the proportion of pediatric providers accepting current or new Medicaid- or CHIP-covered pediatric patients could account for new disparities or the elimination of previous disparities in health and dental insurance coverage, health status, access to care, and use of services.

### Study limitations and strengths

Certain study limitation should be noted. Although the 2007 NSCH data are the most recently available NSCH data set, secular trends in disparities between 2003 − 2007 may not necessarily generalize to trends in years subsequent to 2008 (part of the 2007 NSCH was conducted in 2008). Certain disparities of limited magnitude could be interpreted as statistical artifacts resulting from multiple comparisons. As noted in the Methods, however, Bonferroni adjustments are not applicable, consistent with published guidelines [11] and prior NSCH disparities analyses [2,3], because a specific a priori hypothesis was tested for each dependent variable. In addition, multivariable adjustment for at least seven relevant covariates theoretically should result in identification of only the most robust disparities, and elimination of any possible statistical artifacts in bivariate analyses. NSCH data are obtained via parental report, so it is possible that parental reports of medical and oral health and healthcare may not necessarily accurately represent similar data abstracted from other sources, such as charts, and intergroup differences in language and culture might have influenced parental willingness to accurately report children’s health or healthcare. Some observed differences might represent normal variations in survey responses or in parents’ knowledge of children’s health status from one wave to another. NSCH does not survey parents about Latino subgroups or limited English proficiency (LEP). Although administered in two languages in 2003 and six languages in 2007, NSCH was not conducted in other less-common languages spoken in the US, so the sampling of LEP parents may not necessarily be generalizable.

Certain study strengths should be noted. This is the first study, to our knowledge, to comprehensively examine secular trends in a wide range of racial/ethnic disparities in children’s oral and medical health and healthcare in all four major US racial/ethnic minority groups and multiracial children. Accessing the non-public NSCH data sets resulted in examination of disparities for all four major racial/ethnic minority groups, as well as for multiracial children. The combined sample size of the two waves is approximately 194,000, and sampling weights allow generalization to the entire non-institutionalized population of US children.

### Implications for practice, research, and policy

#### Data collection, monitoring, and public disclosure

The study findings have several implications for clinical practice, research, and policy. The lack of significant change over time in the total number of specific disparities and of indicators for which at least one racial/ethnic group experience disparities, together with the appearance of 10 new disparities, suggest that racial/ethnic data (as self-identified by the parent) routinely should be collected on all children by health systems, practices, Medicaid, CHIP, managed-care organizations, and private insurers, so that racial/ethnic disparities can be identified, monitored, and targeted as part of quality-improvement efforts. This recommendation is consistent with two recent reports by the Institute of Medicine [17,22], proposals by disparities experts [23,24], and Section 4302 of the Affordable Care Act [25,26]. The persistence and new occurrence over time of racial/ethnic disparities in children’s health and healthcare also indicate that disparities monitoring and public disclosure at least annually should be considered by Medicaid, CHIP, states, counties, health plans, hospitals, and clinics [27]. In addition, the large number of pediatric disparities identified in this analysis indicates that there is an urgent need to expand the number of children’s disparities assessed in periodic disparities reports; for example, only six pediatric disparities indicators were examined in the most recent Agency for Healthcare Research and Quality’s National Healthcare Disparities Report [28], a number which easily could be augmented by incorporating some or all of the 34 indices examined in our study.

#### Insurance coverage, medical/dental homes, quality, and specialty care

Although racial/ethnic minorities comprise 45% of US children, they account for 62% of our nation’s uninsured children [14]. Study findings documenting that Latinos, AIANs, and African-Americans are significantly more likely to be uninsured and sporadically insured than white children underscore the need to provide continuous health-insurance coverage to all children in America. The results showing that multiple disparities exist and have persisted in lack of a personal doctor or nurse and in unmet dental needs highlight the necessity of developing effective interventions and policies for ensuring that every child has a medical and dental home. The prevalence and persistence of children’s disparities also suggests that it may be useful to frame disparities as a quality-of-care issues, as has been pointed out by experts [16] and in a recent IOM report [17]. Although it has been shown that children who need and receive care from a specialist have significantly fewer ED visits and hospitalizations and a greater likelihood of care that is consistent with national practice guidelines than children who do not receive specialty care [29,30], our study finding revealed that minority children are significantly more likely that white children to have problems getting specialty care, indicating that interventions and polices are needed to ensure that all children have access to and receive needed specialty care.

#### Workforce diversity, disparities research, and innovative disparities solutions

The study findings of multiple disparities in children’s health and healthcare and their persistence over time also suggest a need to diversify the healthcare workforce, to conduct more research on children’s disparities, and to develop and implement innovative solutions to eliminate children’s disparities. Even after adjusting for income, communities with high proportions of minorities are four times more likely than other communities to have physician shortages, but underrepresented minority (URM) pediatricians and other physicians are significantly more likely than their non-URM counterparts to care for minority, publicly insured, and uninsured patients [31,32], indicating that diversifying the healthcare workforce might be a powerful mechanism for reducing children’s disparities. A more diverse pediatric workforce might prove particularly useful in addressing disparities identified in this study that might be particularly responsive to greater workforce diversity, including having a personal doctor or nurse, the PCP spending enough time with the child (especially where language barriers are prevalent), receiving all needed medical and dental care, and improving access to specialist and mental healthcare (Table 5). The study results, together with a recent systematic review [4], indicate that more research funding and studies are needed on children’s disparities; for example, only five of 103 studies in the IOM’s report on healthcare disparities specifically addressed children’s disparities [33]. In particular, our study findings suggest that it would be beneficial to fund more research on why there are so many disparities in the medical and oral health and healthcare of children, and the reasons for the persistence of these disparities over time. The study findings—which dramatically underscore the multiplicity, spectrum, depth, and persistence of children’s disparities—also highlight the need for innovative, evidence-based solutions to eliminate these disparities. These might include using geographic information systems and other health-information technologies to identify and target communities experiencing the most substantial disparities, and establishing healthcare empowerment zones—providing needed resources, tailored programs, and community-base participatory processes—in areas of need, which have been shown to be effective [34]. Finally, randomized, controlled trials document that racial/ethnic disparities in children’s health and healthcare can be eliminated, using innovative, family-centered, community-based interventions [12,15], suggesting that more federal and state funding is needed for such interventions, and successful interventions should be incorporated into Medicaid and CHIP programs as best practices.

## Conclusions

After adjustment for relevant covariates, multiple racial/ethnic disparities were documented in oral and medical health and healthcare. Despite increases in the numbers of pediatricians in the US, advances in medical care, the introduction of new antibiotics and other medications, and enhancements in the screening and detection of pediatric diseases during the past decade, the study findings revealed that Latino, African-American, and multiracial children are significantly more likely than white children to have suboptimal health, to be overweight, and to be afflicted with asthma. All racial/ethnic minority groups of children, except for multiracial children, are significantly more likely than white children to have teeth in suboptimal condition. Latinos, AIANs, and African-Americans are significantly more likely than whites to be uninsured and sporadically insured, and these disparities persisted between 2003-2007, except that lack of health insurance arose as a new disparity in 2007 for African-American children. Noteworthy disparities were identified in lacking a personal doctor or nurse, insufficient time with the child’s healthcare provider, and problems getting specialty care. Many substantial and noteworthy disparities varied by racial/ethnic group, and awareness of these particular disparities would seem to be crucial in their prevention and monitoring, as well as the development and implementation of interventions to reduce or eliminate these disparities.

Between 2003 and 2007, some noteworthy progress was made in reducing and eliminating certain racial/ethnic disparities in children’s health and healthcare. In contrast, no significant change over time was seen in the number of indicators for which at least one minority group experienced disparities, either overall, or in any of the three subcategories. Furthermore, neither the total number of specific disparities, nor the numbers of specific disparities in the medical/oral health and access to medical and dental care categories changed significantly over time.

Implications of the study findings for practice, research, and policy include: 1) routine data collection, monitoring, and public disclosure should occur for racial/ethnic disparities in children’s health and healthcare by health systems, practices, Medicaid, CHIP, managed-care organizations, and private insurers; 2) interventions and polices are needed to ensure insurance coverage, medical/dental homes, quality of care, and specialty care for all children, regardless of race/ethnicity; and 3) there is an urgent need to diversify the healthcare workforce, conduct more research on children’s disparities, and develop and implement innovative solutions to eliminate disparities in children’s health and healthcare.

## Abbreviations

AIA: American Indian/Alaska Native; API: Asian/Pacific Islander; ED: Emergency department; FPT: Federal poverty threshold; LEP: Limited English proficiency; NSCH: National Survey of Children’s Health; PCP: Primary-care provider; PDN: Personal doctor or nurse.

## Competing interests

The authors declare that they have no competing interests.

## Authors’ contributions

GF conceived of and designed the study, interpreted the data, and wrote the manuscript. HL acquired the data, performed analyses, and contributed to the drafting and revision of the manuscript. All authors reviewed and approved the final manuscript.

## Supplementary Material

Additional file 1 **Appendix 1.** Multivariable analyses^a^ of racial/ethnic disparities in medical and oral health status among US children 0-17 years old (N = 102,353), 2003 NSCH. **Appendix 2**. Multivariable analyses^a^ of racial/ethnic disparities in access to medical and dental care among US children 0-17 years old (N = 102,353), 2003 NSCH. Click here for file
