# Supplementary material for: Trends in racial/ethnic disparities in medical and oral health, access to care, and use of services in US children: has anything changed over the years?
Source: Int J Equity Health. 2013 Jan 22;12:10. doi: 10.1186/1475-9276-12-10 (PMC3560223; doi:10.1186/1475-9276-12-10)
Supplement: Additional file 1 — Appendix 1. Multivariable analysesa of racial/ethnic disparities in medical and oral health status among US children 0-17 years old (N = 102,353), 2003 NSCH. Appendix 2. Multivariable analysesa of racial/ethnic disparities in access to medical and dental care among US children 0-17 years old (N = 102,353), 2003 NSCH. [file 1475-9276-12-10-S1.docx]

**Appendix 1.** Multivariable analyses^a^ of racial/ethnic disparities in medical and oral health status among US children 0-17 years old (N=102,353), 2003 NSCH.

|  | Odds Ratio (95% Confidence Interval) vs. White Children | | | | |
| --- | --- | --- | --- | --- | --- |
| Measure | Latino | **African-American** | Asian/ **Pacific Islander** | Native American | Multiracial |
| Health not excellent/very good | 1.90 (1.67, 2.15) | 1.94 (1.75, 2.14) | 0.99 (0.73, 1.35) | 1.23 (0.9, 1.68) | 1.32 (1.10, 1.59) |
| Overweight or at risk for overweight | 1.38 (1.24, 1.54) | 1.86 (1.71, 2.02) | 0.89 (0.67, 1.2) | 1.67 (1.28, 2.17) | 1.12 (0.97, 1.3) |
| Needs more medical care than others | 1.01 (0.87, 1.18) | 0.81 (0.73, 0.92) | 0.53 (0.34, 0.83) | 1.28 (0.96, 1.7) | 1.12 (0.93, 1.33) |
| Has limited abilities | 0.88 (0.71, 1.09) | 1.29 (1.13, 1.48) | 1.33 (0.77, 2.31) | 1.85 (1.26, 2.72) | 1.34 (1.03, 1.74) |
| Needs/gets special therapy† | 0.97 (0.8, 1.16) | 0.94 (0.8, 1.09) | 0.45 (0.27, 0.75) | 1.56 (1.01, 2.40) | 1.08 (0.85, 1.38) |
| Emotional, developmental, or behavioral  problems needing treatment or counseling | 1.07 (0.89, 1.28) | 0.85 (0.73, 1.0) | 0.50 (0.31, 0.81) | 0.91 (0.62, 1.32) | 1.14 (0.87, 1.48) |
| Learning disability | 1.1 (0.94, 1.3) | 0.91 (0.79, 1.04) | 0.33 (0.22, 0.52) | 1.1 (0.8, 1.5) | 1.12 (0.9, 1.4) |
| Asthmatic | 1.34 (1.16, 1.54) | 1.60 (1.45, 1.78) | 1.25 (0.9, 1.75) | 1.43 (1.07, 1.91) | 1.38 (1.17, 1.63) |
| Hearing/vision problems | 0.95 (0.7, 1.29) | 0.54 (0.42, 0.70) | 0.66 (0.2, 2.19) | 1.97 (1.19, 3.25) | 0.97 (0.67, 1.4) |
| ADHD | 0.73 (0.58, 0.91) | 0.71 (0.61, 0.83) | 0.52 (0.28, 0.97) | 0.74 (0.5, 1.09) | 1.04 (0.79, 1.37) |
| Depression/anxiety | 1.04 (0.82, 1.32) | 0.49 (0.39, 0.61) | 0.31 (0.14, 0.66) | 1.15 (0.74, 1.81) | 1.14 (0.83, 1.57) |
| Behavior problems | 0.93 (0.73, 1.18) | 1.21 (1.01, 1.42) | 0.41 (0.21, 0.80) | 0.86 (0.56, 1.31) | 1.32 (0.98, 1.77) |
| Bone/joint/muscle problems | 0.95 (0.76, 1.2) | 0.91 (0.73, 1.12) | 0.27 (0.15, 0.48) | 1.48 (0.92, 2.37) | 1.26 (0.93, 1.71) |
| Diabetes | 0.79 (0.38, 1.63) | 0.55 (0.33, 0.91) | 0.28 (0.05, 1.63) | 1.35 (0.51, 3.55)‡ | 0.8 (0.43, 1.52) |
| Developmental delay | 0.81 (0.64, 1.02) | 0.67 (0.55, 0.83) | 0.53 (0.35, 0.82) | 0.98 (0.66, 1.44) | 1.1 (0.81, 1.49) |
| Digestive allergies | 1.12 (0.85, 1.47) | 1.01 (0.84, 1.22) | 1.06 (0.73, 1.56) | 0.93 (0.6, 1.46) | 1.57 (1.20, 2.06) |
| Skin allergies | 1.08 (0.92, 1.26) | 1.82 (1.64, 2.02) | 1.16 (0.82, 1.63) | 1.38 (1.01, 1.88) | 1.59 (1.34, 1.90) |
| Speech problems | 1.06 (0.79, 1.43) | 1.35 (1.10, 1.66) | 0.69 (0.32, 1.49) | 1.73 (0.81, 3.67) | 1.13 (0.81, 1.58) |
| ≥ 3 ear infections in last 12 months | 0.73 (0.57, 0.93) | 0.67 (0.55, 0.82) | 0.37 (0.17, 0.81) | 1.03 (0.62, 1.73) | 0.94 (0.69, 1.27) |
| Teeth condition not excellent/very good | 1.64 (1.47-1.81) | 1.63 (1.51-1.77) | 1.33 (1.03, 1.71) | 1.19 (0.94, 1.5) | 1.37 (1.19-1.58) |

Data source: non-public data set of 2003National Survey of Childhood Health.

*Adjusted for primary language spoken at home, child’s age and insurance coverage, caregiver’s educational attainment and employment status, number of children in the household, number of adults in the household, and poverty level

†Includes physical, occupational, or speech therapy

‡Non-significant trend, with *P*=.542

**Appendix 2.** Multivariable analyses^a^ of racial/ethnic disparities in access to medical and dental care among US children 0-17 years old (N=102,353), 2003 NSCH.

|  | Odds Ratio (95% Confidence Interval) vs. White Children | | | | |
| --- | --- | --- | --- | --- | --- |
| Measure | Latino | **African-American** | Asian/ **Pacific Islander** | Native American | Multiracial |
| No health insurance^b^ | 1.50 (1.28, 1.74) | 1.16 (0.99, 1.36) | 0.53 (0.34, 0.82) | 1.73 (1.30, 2.30) | 1.31 (1.01, 1.69) |
| Sporadically insured in past year^b^ | 1.38 (1.19, 1.59) | 1.41 (1.26, 1.59) | 0.97 (0.68, 1.37) | 1.77 (1.34, 2.34) | 1.42 (1.18, 1.70) |
| No personal doctor or nurse | 1.79(1.58, 2.02) | 1.99 (1.81, 2.20) | 1.16 (0.81, 1.66) | 2.72 (2.08, 3.56) | 1.37 (1.14, 1.65) |
| PCP never/sometimes spends enough time with you | 1.94 (1.73, 2.19) | 2.59 (2.36, 2.85) | 2.35(1.85, 2.98) | 1.94 (1.51, 2.51) | 1.2(1.00, 1.43) |
| Did not receive all needed medical care | 1.07 (0.74, 1.55) | 1.51 (0.9, 2.53) | 0.73 (0.27, 2.0) | 4.43 (2.04, 9.64) | 2.83 (1.78, 4.51) |
| Did not receive all needed dental care | 1.27 (0.91, 1.76) | 2.09 (1.62, 2.69) | 1.38 (0.63, 3.05) | 1.85 (1.08, 3.19) | 2.41 (1.55, 3.74) |
| Any problem getting specialty care | 1.34 (1.06, 1.70) | 1.07 (0.86, 1.32) | 2.21 (1.28, 3.80) | 2.37 (1.26, 4.46) | 1.23 (0.91, 1.66) |
| No physician visit in last 12 months | 1.19 (1.03, 1.37) | 1.30 (1.17, 1.45) | 2.19 (1.61, 2.97) | 1.58 (1.18, 2.12) | 1.26 (1.02, 1.55) |
| No routine preventive dental visit in last 12 months | 1.34 (1.06, 1.70) | 1.95 (1.67, 2.27) | 1.72 (0.997, 2.96) | 2.54 (1.53, 4.21) | 1.40 (1.03, 1.90) |
| Received no specialty care in last 12 mos | 1.22 (1.09, 1.36) | 1.46(1.33, 1.61) | 1.84(1.42, 2.39) | 1.33(0.97, 1.84) | 0.89 (0.77, 1.03) |
| Received no mental healthcare in past 12 months | 1.08 (0.9, 1.29) | 1.73 (1.46, 2.06) | 2.47 (1.52, 4.02) | 1.05 (0.71, 1.56) | 0.85 (0.66, 1.1) |

Data source: non-public data set of 2003National Survey of Childhood Health.

^a^Except as noted below, adjusted for primary language spoken at home, child’s age and health insurance coverage, caregiver’s educational attainment and employment status, number of children in the household, number of adults in the household, and poverty level.

^b^Adjusted for primary language spoken at home, child’s age, caregiver’s educational attainment and employment status, number of children in the household, number of adults in the household, and poverty level
